# Determination of dimethylsulfoniopropionate and dimethylsulfoxide in *Posidonia oceanica* leaf tissue

**DOI:** 10.1016/j.mex.2018.12.014

**Published:** 2018-12-27

**Authors:** W. Champenois, A.V. Borges

**Affiliations:** Chemical Oceanography Unit, University of Liège, Belgium

**Keywords:** Determination of dimethylsulfoniopropionate and dimethylsulfoxide in *Posidonia oceanica* tissues by gas chromatography, Dimethylsulfoniopropionate, Dimethylsulfoxide, *Posidonia oceanica*

## Abstract

In order to investigate the possible use of the dimethylsulfoniopropionate (DMSP) and dimethylsulfoxide (DMSO) ratio as a stress indicator of *Posidonia oceanica* a method for the determination of these quantities was developed for this type of material.

• The method relies on gas chromatography with headspace technique, instead of the purge-and-trap technique commonly used.
• The method allows the determination of both DMSP and DMSO on the same sample.
• This method allows to quantify DMSP, DMSO and DMSP:DMSO ratio for calibration curves with a coefficient of variation around 2% and a relative error around 2% and within the ranges natural variability of DMSP and DMSO in *P. oceanica* leaf tissue.

Preliminary tests showed that DMSP in *P. oceanica* leaf tissue ranged from 20 to 200 μmol g^−1^ of fresh weight (FW) and 2 to 5 μmol g_fw_^−1^ for DMSO. The DMSP:DMSO ratio ranged from 2 to 40. The quantifications were conducted with different mixtures of DMSP and DMSO by measurements of DMSP and DMSO in the same sample of *P. oceanica* leaf tissue.

## Graphical abstract

**Figure:**
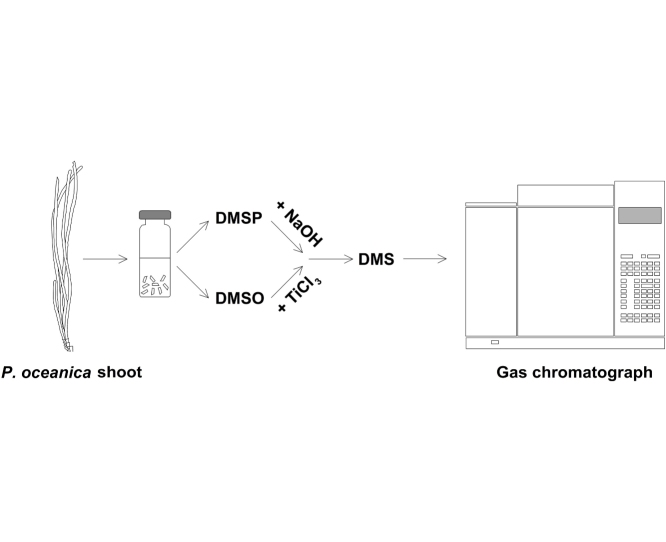

**Specifications Table**

**Table:** 

Subject Area	• *Environmental Science*
More specific subject area:	*Biogeochemistry of dimethylsulfoniopropionate and dimethylsulfoxide in seagrass*
Method name:	*Determination of dimethylsulfoniopropionate and dimethylsulfoxide in Posidonia oceanica tissues by gas chromatography*
Name and reference of original method	*Stefels, J., 2009. Determination of DMS, DMSP, and DMSO in seawater. In: Wurl, O. (Ed.), Practical Guidelines for the Analysis of Seawater, 223–234.*
Resource availability	*Not applicable*

## Method details

*Posidonia oceanica* is an endemic keystone specie of the Mediterranean coast. This macrophyte is able to produce dimethylsulfoniopropionate (DMSP) [1,2]. The role of DMSP in the *P*. *oceanica* remains unclear. Otte et al [3] listed different roles for DMSP in macrophytes: methylation, detoxification of sediment by removal of sulphide and reduction of overflow of energy, cryoprotectant or osmoregulation. In the case of *P*. *oceanica*, DMSP cannot act as osmolyte because this macrophyte is stenohalin. Similarly, DMSP cannot act as a cryoprotectant because *P*. oceanica is endemic of the Mediterranean Sea where water never freezes. DMSP is the precursor of dimethyl sulfide (DMS) and acrylic acid; these two compounds can act as herbivore deterrent for macrophytes [3]. DMSP acts as antioxidant in marine phytoplankton [4] and higher plants such as *Spartina alterniflora* [5], and in both cases DMSP is oxidized to dimethylsulfoxide (DMSO). Mcfarlin and Alber [6] suggested that the DMSO:DMSP ratio can be used as an indicator of stress of *S. alterniflora* for a variety of disturbances. The aim of this paper is to describe a method to accurately measure the DMSP:DMSO ratio in *P. oceanica* tissues with the possible goal of using this ratio as a stress indicator for this macrophyte by analogy with *S. alterniflora.* The method we propose here is an adaptation of DMS(P,O) analytical method that is used typically for phytoplankton [7], that was adapted for *P*. *oceanica* leaf tissue, and is based on the headspace technique, instead of purge-and-trap.

### Experimental set-up

We have shown that *P. oceanica* leaf tissues can have a high concentration of DMSP (up to 200 μmol g^−1^ of fresh weight (fw)) and DMSO (up to 2 μmol g_fw_^−1^) [2]. DMSP is quantitatively cleaved into DMS and acrylate in a NaOH solution and DMSO is quantitatively reduced in DMS with TiCl_3_ in an acid solution [7]. To determine the concentration of dissolved DMS it is possible to use the headspace technique. Normally, to use the headspace technique the Henry’s constant (HC) should be known. With the knowledge of HC and the partial pressure of DSM in the gas phase it is possible to compute the DMS dissolved concentration in the aqueous phase. But the HC depends on several of parameters (mainly temperature and ionic strength). Similarly to [6], we used another approach based on the measurement of the DMS concentration in the headspace and to treat the samples under the same conditions as the standards (head space and liquid volumes, concentration of the solutions, and temperature). Also, we used the same vial for the determination of DMSP and DMSO.

### DMSP and DMSO calibration curves

The calibration curves were built with solutions prepared from pure DMSP (Research Plus Inc) and pure DMSO (Analytic grade, Merck). The preparation of the two standard solutions was exactly the same. We prepared gravimetrically a stock solution (SS) with a concentration around 2 10^−3^ mol L^−1^. For DMSP, the weigh-in was transferred to a 50 ml graduated flask and filled with HCl (10^−2^ mol L^−1^). For DMSO, the weigh-in was transferred to a 50 ml graduated flask and filled with ultrapure (Type 1) Milli-Q water. We prepared a working solution (WS) with an adequate volume of SS. This volume was taken from the SS and transferred to a 50 ml graduated flask and filled with ultrapure (Type 1) Milli-Q water. The standards were made by mixing correct volumes of the two WS to have always 2.5 ml in total (Table 1). These volumes were transferred to borosilicate 20 ml vials to which was added 2.5 ml of NaOH solution (12 mol L^−1^) with the purpose of cleaving DMSP into DMS and acrylate. The vials were immediately sealed with a gas-tight polytetrafluoroethylene coated silicone septa. Three replicates were prepared for each concentration. After at least 24 h at room temperature (close to 25 °C), a volume of 100 μl of gas was sampled with a 100 μl Hamilton gas-tight syringe from the headspace, and was injected directly to the head of chromatographic column of an Agilent 7890b gas chromatograph (CG) fitted with a flame photometric detector (FPD) for the determination of DMSP concentration. The FPD was kept at 250°C with H_2_ and synthetic air flows (respectively 50 and 60 ml min^−1^) (Air Liquide Belgium). The column was a capillary column (CP-Sil 5CB, 30 m long, 0.32 mm internal diameter, 0.5 μm film thickness, Chromatographie Service GmbH) the carrier gas was ultrapure He (2 ml min^−1^) (Air Liquide Belgium, alphagas-2 grade). The temperature of the oven was kept at 60 °C. It should be noted that the cleavage reaction is known to be complete within 15 min [8], nevertheless in order to digest the cell walls of *P. oceanica* leaves, we have adopted reaction time of at least 24 h. Prior to the determination of DMSO, the DMS produced by the cleavage of DMSP was stripped by gentle bubbling of compressed air during 20 min through the septa. We used compressed air to strip DMS because it is easier and cheaper than a pure gas cylinder (N_2_ ou He) that can also be used. The compressor is located way from our laboratory (elsewhere in the building) so direct contamination from the atmosphere of our laboratory is not possible. This was nevertheless tested, and the absence of DMS/DMSP/DMSO in compressed air is proven by undetectable DMS peaks in the blanks (DMSP and DMSO). Afterwards, we removed the septa and we added 2.5 ml of pure HCl (12 mol L^−1^) (HCl 37% Normapur, VWR) to acidify the solution. We then added 1ml of TiCl_3_ (30%, Merck) to reduce DMSO into DMS, and we immediately sealed the vial with a gas-tight polytetrafluoroethylene coated silicone septa. At the room temperature the reaction rate is not fast therefore we prefered to add exactly 1ml of TiCl3 (with a micropipette) before sealing. Nevertheless, even assuming a reduction efficiency <100%, this should have not been a problem since the systems is calibrated against DMSO standards, and the reduction efficiency is assumed to be the same for both standards and samples. After at least 24 h at room temperature, we injected 1 ml of NaOH (12 mol L^−1^) through the septa in order to remove the fumes of HCl, and protect the GC from corrosion. The addition of NaOH leads to a warming of the solution, so it is advisable to wait for the vials to cool to room temperature (about 30 min). We then brought the headspace to the atmospheric pressure by removing the overpressure of the headspace by a rapid introduction of a hypodermic needle through the septa. In order to make sure that the gas phase and aqueous phase were in equilibrium, we waited at least 30 min during which time the vials were shaken twice. Then, 100 μL of gas headspace were injected into the CG at the head of the column through split-splitless injection port. It should be noted that the blank for DMSP (or DMSO) corresponding to higher concentration on DMSO (or DMSP) (Table 1). The fact that in the DMSO blank we never detected a DMS peak proves that DMS produced from DMSP was completely removed.

**Table 1:** Example of dilution for DMSP and DMSO strands for calibration curves as given in Fig. 1, made from stock solutions of 2.04 10^−3^ mol L^−1^ for DMSP and 2.18 10^−3^ mol L^−1^ for DMSO, and working solutions of 2.04 10^−4^ mol L^−1^ for DMSP and 2.18 10^−5^ mol L^−1^ for DMSO, where C* correspond to the different concentrations of DMSP and DMSO.

	C1	C2	C3	C4	C5	C6	C7	C8	C9	C10	C11
Vol DMSP (ml)	0	0.25	0.50	0.75	1.00	1.25	1.50	1.75	2.00	2.25	2.50
Vol DMSO (ml)	2.50	2.25	2.00	1.75	1.50	1.25	1.00	0.75	0.50	0.25	0
Vol NaOH (ml) 12M	2.50	2.50	2.50	2.50	2.50	2.50	2.50	2.50	2.50	2.50	2.50
Vol Tot (ml)	5.00	5.00	5.00	5.00	5.00	5.00	5.00	5.00	5.00	5.00	5.00
Conc DMSP (μmol L^−1^)	0	10.2	20.4	30.6	40.8	51.0	61.2	71.4	81.6	91.8	102
Conc DMSO (μmol L^−1^)	21.9	19.7	17.5	15.3	13.1	11.0	8.76	6.57	4.38	2.19	0

### Sample collection, preservation and preparation of *P. oceanica* leaves

The collection, preservation and preparation of sample of *P. oceanica* leaves are described in detail elsewhere [1,2]. In brief, *P. oceanica* leaves were collected (in triplicates) by SCUBA diver and directly frozen at −20 °C after removal of epiphytes by scrapping with a razor blade [9]. We uses the 10 cm section from the bottom of the leaf. In the home laboratory, the samples were removed from the freezer and carefully dried of defrost water with absorbing paper. The piece of the leaf was immediately cut with stainless steel scissors into square pieces (3 × 3 mm) and mixed together. We weighted around 20 mg of the mix in pre-weighted borosilicate (20 ml) vials to which we added 2.5 ml of Milli-Q water. We added 2.5 ml of the same solution of NaOH (12 M) as for the standards. This takes typically no more than one minute or two, so we assume the loss of water from the tissues to be minimal. The sample added around 0.05% of ionic strength with respect of standard. We have considered this addition as insignificant with regards to change in Henry’s constant and assumption that phase equilibrium in the headspace follows the same solubility for samples and standards. The vials were immediately sealed. From here, standards and sample undergo the same procedures. To test if free DMS in the fresh weight samples can interfere with DMSP or DMSO quantification we conducted the following test: more or less 100 mg of *P. oceanica* leave tissue (5 times more than normally) were put in 20 ml vial with 5 ml of Milli-Q water and the vial was sealed (in triplicate). In order to open the cell walls and release possibly enclosed DMS, the vials were exposed to 2 h of ultrasound sonication. 100 μl of the headspaces (10 times more than normally) were sampled and injected into the GC. In all three replicates, DMS content (peak) was non-quantifiable (below detection limit). We conclude that the DMS present in the tissue was at levels that do not interfere with DMSP or DMSO quantification in *P. oceanica* leaves.

Statistical analysis was made with Graphpad Prism3.

## Method validation

**Fig. 1:**
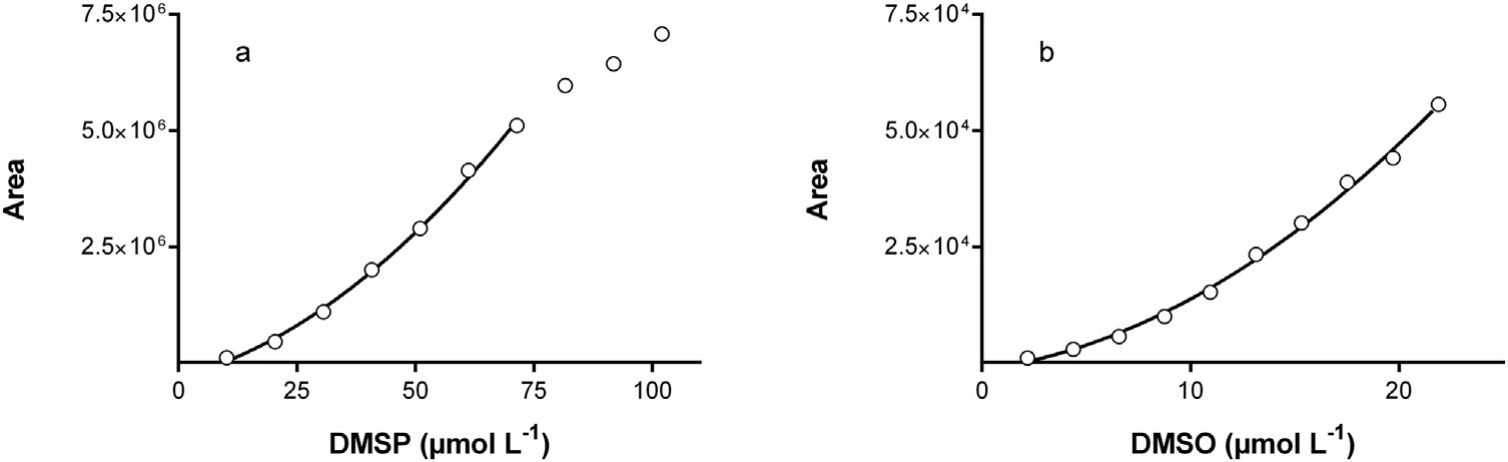
Calibration curves for the dilutions given in Table 1. On the left panel (a) the response of FPD with DMSP concentration is clearly sigmoidal. By limiting the DMSP concentration range from 10 to 80 μmol L^−1^ it is possible to fit a polynomial second order model (line). On the right panel (b) the response of FPD with the DMSO concentration range can be fit with fit a polynomial second order model (line).

Fig. 1 show the calibration curves for DMSP and DMSO based on the mixtures of DMSP and DMSO given in Table 1. We can see on Fig. 1a that the response of the FPD to the DMS concentration is sigmoidal. If we restrict the calibration to DMS concentration from 0 to 80 μmol L^−1^ (n = 7), we can use a polynomial second order model to fit the calibration curve, with a determination coefficient (R^2^) of 0.989. For DMSO (Fig. 1b), we also used a polynomial second order model (R^2^ = 0.996, n = 9) to fit the calibration curve. The blank for DMSP gave zero of DMS concentration. Since it is possible to fit a polynomial second order model to the DMSP data, we considered that there was no interferences of the huge presence of DMSO in the DMSP standards. In the same way, the removal of DMS from the DMSP determination prior to the DMSO determination seemed to be complete since the DMSO blank gave zero. It was possible to measure, independently, DMSP and DMSO in the same vial.

**Fig. 2:**
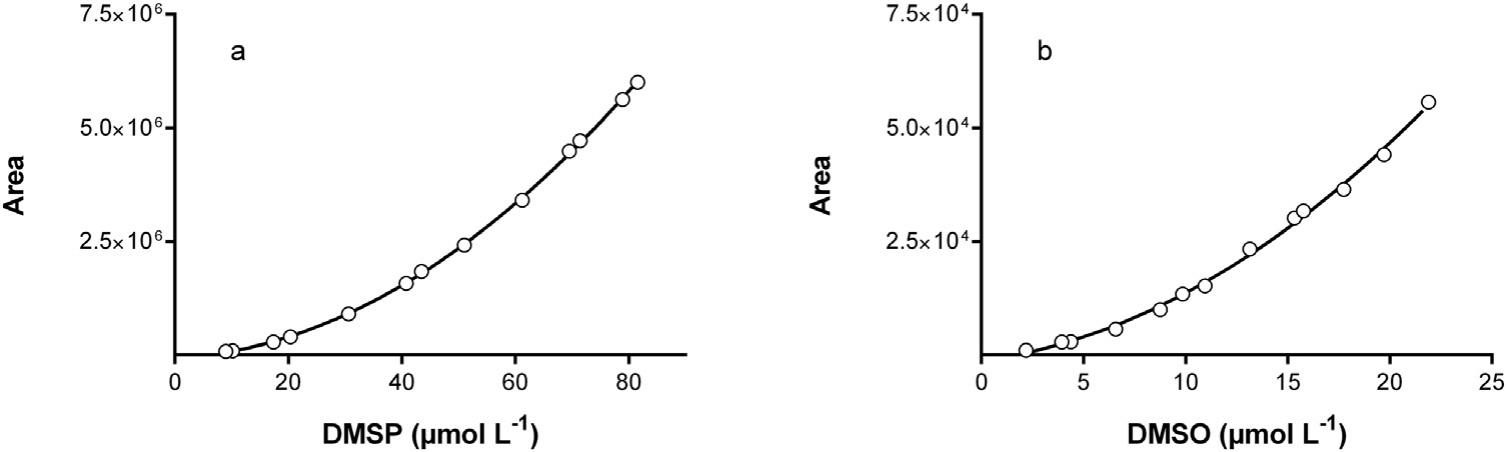
Complete calibration curves for the dilutions shown in Table 2.

On Fig. 2, we present an example of complete calibration curves for DMSP and DMSO that were fitted with second order polynomial model curves. The main characteristics of the calibrations curves are given in Table 2. With second order polynomial model curves we computed the modeled concentration of the DMSP (O) for each vial (three by concentration). We computed coefficient of variation (CV) from the modeled concentrations. The relative error (RE) was computed between real concentration and the average of each modeled concentrations. In order to compare the concentrations in the vials and in *P. oceanica* leaf tissue, we add equivalent concentration of DMSP(O) in one gram of fresh weigh. This approximation was made and we obtained a theoretical weight of 0.020 g of sample.

**Table 2:** Complete standard curves and the main characteristics of this standardization.

Real DMSP concentration (μmol L^−1^)	8.98	10.20	17.38	20.40	30.60	40.80	43.45	51.00	61.20	69.52	71.40	78.90	81.60
Modelled DMSP concentration (μmol L^−1^)	9.81	10.50	17.04	19.95	30.35	40.52	44.00	50.80	60.65	70.01	71.80	78.70	81.40
Standard deviation DMSP (μmol L^−1^)	0.06	0.13	0.18	1.03	0.60	3.31	2.83	0.85	0.19	2.25	2.98	1.24	0.90
CV% DMSP	0.62	1.21	1.07	5.15	1.97	8.18	6.42	1.68	0.31	3.21	4.15	1.58	0.01
Relative error (%) DMSP	−9.22	−2.89	1.95	2.19	0.82	0.68	−1.25	0.40	0.89	−0.70	−0.56	0.26	0.25
Equivalent μmol DMSP g ^−1^ (FW)	2.25	2.55	4.35	5.10	7.65	10.20	10.86	12.75	15.30	17.38	17.85	19.73	20.40
Real DMSO concentration (μmol L^−1^)	2.19	3.94	4.38	6.57	8.76	9.86	10.95	13.14	15.33	15.77	17.74	19.71	21.9
Modelled DMSO concentration (μmol L^−1^)	2.70	4.20	4.25	6.07	8.35	9.84	10.59	13.47	15.55	15.99	17.28	19.58	21.80
Standard deviation DMSO (μmol L^−1^)	0.04	0.06	0.14	0.04	0.12	0.82	0.22	0.20	0.13	0.21	0.49	0.12	0.11
CV% DMSO	1.60	1.34	3.32	0.58	1.42	8.33	2.05	1.45	0.87	1.33	2.81	0.59	0.50
Relative error (%) DMSO	20.90	7.45	−1.00	−7.03	−5.97	4.88	−1.38	1.80	1.93	2.88	−0.30	−0.24	−0.60
Equivalent μmol DMSO g ^−1^ (FW)	0.55	0.99	1.10	1.64	2.19	2.46	2.74	3.29	3.83	3.94	4.43	4.93	5.48

For DMSP (Fig. 2a), the equation of the calibration curve was:

Area = 837.2 C_DMSP_^2^ +6498.6 C_DMSP_ -74227 (n = 13, R^2^ = 0.9997)

where C_DMSP_ is the DMSP concentration.

We can see (Table 2) that the averages of CV and RE were respectively 2.7% and 1.7% for the full range of DMSP concentrations. The high CV can be related to the manual injection for example the two high CV 8.18% and 6.42% for respectively 40.80 and 43.45 μmol L^−1^ on DMSP are related at low RE (0.68% and −1.25%). The limit of quantification was estimated around the 10 μmol L^−1^ corresponding to 2.5 μmol DMSP g_fw_^−1^. Such high limit is not a problem because de DMSP concentration in *P. oceanica* leaf is larger [2]. Outside the range, we can use this method for concentrations up to 80 μmol L^−1^ which means 20.4 μmol DMSP g_fw_^−1^. We have shown that the DMSP concentration in *P. oceanica* leaf tissue can be as high as 200 μmol g_fw_^−1^ which corresponds to a concentration of 800 μmol L^−1^. Since it is not realistic to use 0.002 g_fw_ of sample, we decided to inject 10 μl of headspace gas into the CG and multiply the result of concentration by 10. In the order to verify this procedure, we prepared three (each in triplicate) different concentrations in DMSP of 204, 408 and 816 μmol L^−1^. The three concentrations corresponded to 51, 102 and 204 μmol DMSP g_fw_^−1^. We injected into the CG 10 μl of gas from the headspace with a gas-tight Hamilton syringe of 10 μl. The results were 203, 409 and 815 μmol L^−1^, respectively, with an average CV of 1.0% and a RE < 1%.

For DMSO (Fig. 2b) the equation of the calibration curve was:

Area = 91.2 C_DMSO_^2^ + 573.0 C_DMSO_ −1262 (n = 13, R^2^ = 0.9971).

where C_DMSO_ is the DMSO concentration.

The averages of CV and RE were, respectively, 2.1% and 4.6% for all the range (Table 2). It should be noted that the highest RE (20.9% and 7.4%) were for the two lower concentrations. With a range from 4.38 to 21.9 μmol L^−1^ the RE was 2.30%. The limit of quantification was around 4 μmol L^−1^ which is equivalent to 1 μmol DMSO g_fw_^−1^. The concentration of DMSO in *P. oceanica* leaf tissue is larger than this limit of quantification, typically around 2 μmol DMSO g_fw_^−1^ (FW) [2]. For the roots and the rhizome, first tests have shown that the DMSO concentrations are weaker (1 μmol DMSO g_fw_^−1^), and in this case it is advisable to use more sample material.

**Fig. 3:**
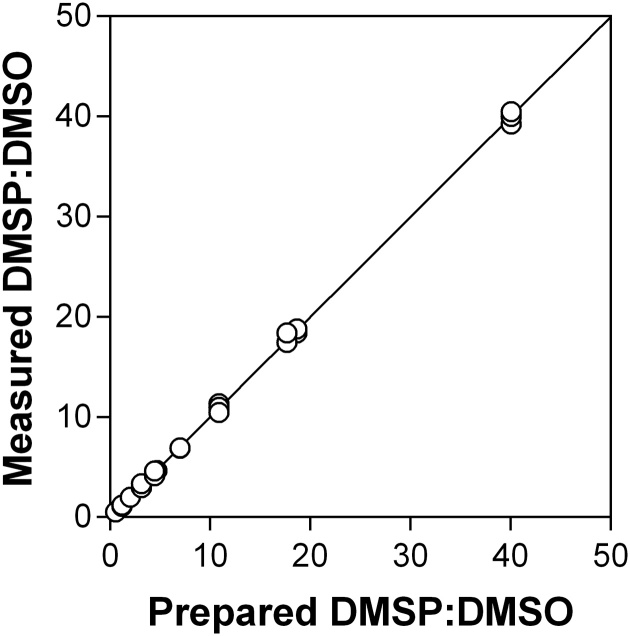
Comparison between the prepared DMSP:DMSO ratio and the measured DMSP:DMSO ratio.

As a final test of the procedure, we prepared different mixtures of DMSP and DMSO covering the range of 20–200 μmol DMSP g_fw_^−1^ for DMSP and 1–6 μmol DMSO g_fw_^−1^ for DMSO, corresponding to a DMSP:DMSO ratio from 0.5 to 40. It should be noted that these ranges of DMSP and DMSO and DMSP:DMSO correspond to the practical concentrations in the *P. oceanica* leaf tissues. Fig. 3 presents the result for 13 different preparations of ratios in triplicate. The slope is 0.9948 ± 0.0037 and the Y-intercept is 0.0127 ± 0.0052 (R^2^ = 0.9995; n = 39). The CV% for the ratios were 2.1%, and we conclude that it is possible to measure DMSP:DMSO ratio with a RE of nearly 2%.
